# Cellular pharmacology of a liposomal preparation of N4-hexadecyl-1-beta-D-arabinofuranosylcytosine, a lipophilic derivative of 1-beta-D-arabinofuranosylcytosine.

**DOI:** 10.1038/bjc.1995.185

**Published:** 1995-05

**Authors:** D. H. Horber, H. Schott, R. A. Schwendener

**Affiliations:** Department of Internal Medicine, University Hospital, Switzerland.

## Abstract

The in vitro deamination, cytotoxicity, cellular drug uptake, distribution and cellular pharmacology in HL-60 cells of N4-hexadecyl-1-beta-D-arabinofuranosylcytosine (NHAC), a lipophilic derivative of arabinofuranosylcytosine (ara-C), were studied. Compared with ara-C, NHAC in liposomal formulations was highly resistant to deamination, resulting in levels of formation of arabinofuranosyluracil 42 and ten times lower in plasma and liver microsomes respectively. The cytotoxicity of NHAC was independent of both the nucleoside transporter mechanism and the deoxycytidine (dCyd) kinase activity as demonstrated by co-incubating NHAC with dipyridamole and/or dCyd. In ara C-resistant HL-60 cells NHAC was still cytotoxic, requiring drug concentration only 1.6 times higher than sensitive cells. Uptake of NHAC was six times higher and was not inhibited by dipyridamole. The pharmacokinetics of NHAC revealed that its intracellular half-life is 4.8 times longer than that of ara-C. Ara-CTP formation and incorporation into DNA was up to 25-50 times lower than that of ara-C and contributed only marginally to the cytotoxic effects of NHAC. These results indicate that, because of the significantly increased stability, the transporter-independent uptake and the dCyd-kinase-independent cytotoxicity, NHAC might be active in ara-C-resistant cells.


British Journal of Cancer (1995) 71, 957–962

© 1995 Stockton Press All rights reserved 0007-0920/95 $12.00

### Cellular pharmacology of a liposomal preparation of $N^4$-hexadecyl-1-β-D-arabinofuranosylcytosine, a lipophilic derivative of 1-β-D-arabinofuranosylcytosine

DH Horber[1], H Schott[2] and RA Schwendener[1]

[1]*Department of Internal Medicine, Medical Oncology, University Hospital, CH-8091 Zürich, Switzerland;* [2]*Institute of Organic Chemistry, University of Tübingen, D-72076 Tübingen, Germany.*

**Summary** The *in vitro* deamination, cytotoxicity, cellular drug uptake, distribution and cellular pharmacology in HL-60 cells of $N^4$-hexadecyl-1-β-D-arabinofuranosylcytosine (NHAC), a lipophilic derivative of arabinofuranosylcytosine (ara-C), were studied. Compared with ara-C, NHAC in liposomal formulations was highly resistant to deamination, resulting in levels of formation of arabinofuranosyluracil 42 and ten times lower in plasma and liver microsomes respectively. The cytotoxicity of NHAC was independent of both the nucleoside transporter mechanism and the deoxycytidine (dCyd) kinase activity as demonstrated by co-incubating NHAC with dipyridamole and/or dCyd. In ara C-resistant HL-60 cells NHAC was still cytotoxic, requiring drug concentration only 1.6 times higher than sensitive cells. Uptake of NHAC was six times higher and was not inhibited by dipyridamole. The pharmacokinetics of NHAC revealed that its intracellular half-life is 4.8 times longer than that of ara-C. Ara-CTP formation and incorporation into DNA was up to 25–50 times lower than that of ara-C and contributed only marginally to the cytotoxic effects of NHAC. These results indicate that, because of the significantly increased stability, the transporter-independent uptake and the dCyd-kinase-independent cytotoxicity, NHAC might be active in ara-C-resistant cells.

**Keywords:** $N^4$-hexadecyl-1-β-D-arabinofuranosylcytosine; cellular pharmacology; HL-60 cells; liposomes

1-β-D-Arabinofuranosylcytosine (ara-C) is one of the most effective agents in the treatment of acute myelogenous leukaemia (Keating *et al.*, 1982; Plunkett and Gandhi, 1993). However, its usefulness is limited by its rapid deamination to the biologically inactive metabolite 1-β-D-arabinofuranosyluracil (ara-U) (Ho and Frei, 1971). Thus, to be therapeutically effective, ara-C must be administered either continuously for 5 days (Frei *et al.*, 1969) or as high-dose regimens up to $3\ \mathrm{g\ m^{-2}}$ (Momparler, 1974). In order to avoid or delay the deamination of ara-C *in vivo*, a large number of $N^4$-derivatives of ara-C have been synthesised (Wempen *et al.*, 1968; Kanai and Ichino, 1974; Rosowsky *et al.*, 1982) or ara-C was used in combination therapies with deaminase inhibitors such as tetrahydrouridine (Kreis *et al.*, 1991) or zebularine (Driscoll *et al.*, 1991). Lipophilic ara-C derivatives modified with long-chain fatty acids show strong anti-tumour activity in murine tumour models (Aoshima *et al.*, 1976; Kataoka and Sakurai, 1980; Tsuruo *et al.*, 1980). A related 5′-substituted liponucleotide, cytarabine ocfosfate, has recently been approved for clinical use in Japan (Houlihan *et al.*, 1995). In a previous study we demonstrated that $N^4$-acyl derivatives of ara-C incorporated into the membranes of small unilamellar liposomes are active against murine L1210 leukaemia and B16 melanoma cells at concentrations 2–4 times lower than ara-C (Rubas *et al.*, 1986). However, resistance of the $N^4$-acyl derivative $N^4$-oleyl-1-β-D-arabinofuranosylcytosine to enzymatic deamination to ara-U was only partially achieved and suggested to be still insufficient in a phase I/II study (Schwendener *et al.*, 1989). For this reason we synthesised the $N^4$-alkyl-ara-C derivative $N^4$-hexadecyl-1-β-D-arabinofuranosylcytosine (NHAC) (Schwendener and Schott, 1992). This compound has greater tumour-inhibitory effects than ara-C in the L1210 tumour model at molar drug concentrations 16 times lower. NHAC also exerts a strong cytotoxic effect at single dose schedules, suggesting a long-lasting drug effect.

Correspondence: RA Schwendener, Department of Internal Medicine, Medical Oncology, University Hospital, Rämistrasse 100, CH-8091 Zurich, Switzerland
Received 31 October 1994; revised 23 December 1994; accepted 29 December 1994

A major reason for treatment failure in leukaemia patients is ara-C resistance. Possible mechanisms of ara-C resistance that have been proposed include low levels of deoxycytidine kinase (Chu and Fisher, 1965), increased catabolism by cytidine deaminase (Steuart and Burke, 1971) and a decreased number of nucleoside transport sites (Wiley *et al.*, 1982). The cellular uptake of ara-C is achieved by transporter mediated facilitated diffusion (Plagemann *et al.*, 1978). Because of the lipophilicity of NHAC we postulate a transporter-independent uptake mechanism as well as altered behaviour in cellular pharmacokinetics.

In the present study, we investigated the deamination kinetics of NHAC and ara-C in human plasma and mouse liver microsomes. Furthermore, we evaluated the properties of NHAC compared with ara-C by studying *in vitro* its cytotoxicity in the human myeloid leukaemia cell line HL-60 and, in ara-C-resistant HL-60/ara-C cells, the cellular uptake, the intracellular drug distribution and pharmacokinetics, the rate of ara-CTP formation and incorporation into DNA.

### Materials and methods

### *Drugs*

Ara-C, dipyridamole and 2′-deoxycytidine (dCyd) were purchased from Sigma (Buchs, Switzerland). [5-$^3$H]Ara-C (30 Ci $\mathrm{mmol^{-1}}$) and custom-synthesised [5-$^3$H]NHAC (5.1 Ci $\mathrm{mmol^{-1}}$) were purchased from Amersham (Amersham, UK). For all incubations, ara-C was dissolved in phosphate-buffered saline (PBS; 8 mM sodium phosphate, 1.5 mM potassium dihydrogen phosphate, 0.14 M sodium chloride, 2.6 mM potassium chloride) with trace amounts of [5-$^3$H]ara-C. NHAC was given in a liposomal formulation as described in the Liposome preparation section. NHAC was synthesised as previously described (Schwendener and Schott, 1992). Tetrahydrouridine was a gift from the Drug Development Branch of the National Cancer Institute (Bethesda, MD, USA).

### *Cells*

HL-60 promyelocytic leukaemia cells were obtained from the American Type Tissue Culture Collection (ATCC CCL 240).

The ara-C-resistant HL-60 cells (HL-60/ara-C) were a kind gift from Dr Studzinski, UMD–New Jersey Medical School, Newark, NJ, USA (Kolla and Studzinski, 1994). This HL-60/ara-C subline has been isolated and characterised by Bhalla *et al.* (1984). The cells were grown in RPMI-1640 medium (Gibco, Paisley, UK) supplemented with 10% heat-inactivated fetal calf serum (FCS; PAA-Biologics, Linz, Austria), 100 units $ml^{-1}$ penicillin and 100 $\mu g\ ml^{-1}$ streptomycin in a humidified 5% carbon dioxide atmosphere. The experiments were initiated in logarithmically growing cultures at a density of $3-5 \times 10^5$ cells $ml^{-1}$.

*Liposome preparation*

Small unilamellar liposomes of 100 ± 30 nm mean diameter were prepared by filter extrusion as described by Hope *et al.* (1985). Briefly, lipid mixtures composed of soy phosphatidylcholine (SPC), cholesterol, *D,L*-α-tocopherol and NHAC at a molar ratio of 1:0.2:0.01:0.1 were hydrated with PBS and sequentially filtrated through Nuclepore (Costar, Sterico, Dietikon, Switzerland) filters of decreasing pore size (1 μm, 400 nm, 100 nm). Liposomes without NHAC, termed empty liposomes, were used as control. All preparations (2–40 mg $ml^{-1}$ SPC) were sterile filtered through 0.2 μm filters (Acrodisc, Gelman, Ann Arbor, MI, USA) and stored at 4°C. Trace amounts of [5-$^3$H]NHAC were added for detection and quantification.

*Deamination studies*

Fresh human plasma or freshly prepared mouse liver microsomes were incubated with [5-$^3$H]ara-C (1 μCi per sample) dissolved in PBS or [5-$^3$H]NHAC-liposomes either for different time periods at a concentration of 2 μM or with increasing concentrations (0–1.33 mM) of the drugs for 3 h at 37°C. Microsomal incubations were carried out in the presence of 0.6 mM NADP, 3 mM glucose 6-phosphate, 0.6 units $ml^{-1}$ glucose-6-phosphate dehydrogenase (Boehringer Mannheim, Germany) and 4.6 mM magnesium chloride. To inhibit further deamination, tetrahydrouridine (2 μM) was added after the incubation and the probes were ultrafiltrated using Diaflo YM membranes (Amicon, Lexington, MA, USA; $M_r$ 10 000 cut-off). The filtrates were analysed for ara-U by ion-exchange high performance liquid chromatography (HPLC) on a Partisil SCX column (Knauer, Berlin, Germany) using potassium dihydrogen phosphate (15 mM, pH 2.5) as elution phase at a flow rate of 1.2 ml $min^{-1}$ (Spriggs *et al.*, 1987). The fractions containing ara-U were pooled and quantified by scintillation counting. Protein concentration in the microsomes was determined as described by Bradford (1976). The final protein concentration in the probes was 3.4 mg $ml^{-1}$.

*Cytotoxicity assays*

HL-60 cells were counted and seeded in 96-well plates ($3 \times 10^5$ cells $ml^{-1}$). Then the cells were exposed to various concentrations (0–200 μM) of ara-C, NHAC or empty liposomes in the presence or absence of 20 μM dipyridamole and/or 20 μM dCyd for 24 h at 37°C (5% carbon dioxide). Dipyridamole and/or dCyd were added 5 min before drug exposure. After incubation the medium was removed, the cells washed once and resuspended in fresh, serum-free RPMI-1640 medium. The cell survival fractions were determined with the 3-(4,5-dimethylthiazol-2-yl)-2,5-diphenyltetrazolium bromide (MTT) dye reduction assay as described by Mosmann (1983). All experiments were repeated four times. Accordingly, the cytotoxic effects of ara-C and liposomal NHAC against HL-60/ara-C-resistant cells were measured with the MTT assay but without any additives. Cell growth-inhibitory concentrations ($IC_{50}$ and $IC_{20}$) were calculated from interpolations of the graphical data.

*Drug uptake*

HL-60 cells ($2 \times 10^6$ cells per well) were incubated in 24-well plates with increasing concentrations up to 200 μM of [5-$^3$H]ara-C or [5-$^3$H]NHAC (2 μCi per sample) in the presence or absence of the nucleoside-transport blocking agent dipyridamole (20 μM) for 3 h at 37°C (5% carbon dioxide). Dipyridamole was added 5 min before drug exposure. After washing twice with cold PBS, total drug uptake was determined by scintillation counting. All experiments were performed in triplicate.

*Cellular pharmacokinetics*

HL-60 cells ($5 \times 10^6$ cells per well) were incubated with 2 μM [5-$^3$H]ara-C or [5-$^3$H]NHAC (2 μCi per sample) for 2 h at 37°C (5% carbon dioxide). Cells were washed twice with cold PBS to remove unbound drug. The incubations were continued in RPMI medium and stopped after different time periods up to 3.5 h to determine peak concentration, time-dependent total drug uptake and 1-β-D-arabinofuranosylcytosine triphosphate (ara-CTP) formation. Intracellular half-lives were calculated by linear regression of semilogarithmic concentration *vs* time plots. The area under the curve (AUC) was determined from these plots using proFit software (Quantumsoft, Zurich, Switzerland). All experiments were performed in triplicate.

*Cellular ara-CTP formation*

HL-60 cells ($5 \times 10^6$ cells per well) were incubated with increasing concentrations of [5-$^3$H]ara-C or [5-$^3$H]NHAC (2 μCi per sample) for 2 h at 37°C (5% carbon dioxide). After washing twice with cold PBS, the cells were lysed with 0.4 M perchloric acid and centrifuged after 10 min (10 000 *g* for 2 min). The supernatants were collected, neutralised with 10 M potassium hydroxide and centrifuged (12 000 *g* for 10 min). The resulting supernatant was analysed for ara-CTP by ion-exchange HPLC using a Spherisorb SAX column (Phenomenex, Torrance, CA, USA) and 125 mM potassium dihydrogen phosphate plus 75 mM trisodium citrate (pH 4.6) as elution buffer at a flow rate of 0.45 ml $min^{-1}$. Ara-CTP-containing fractions were pooled and analysed by scintillation counting. All experiments were performed in triplicate.

*Incorporation into DNA*

HL-60 cells ($1.8 \times 10^7$ cells per well) were incubated with 2 μM [5-$^3$H]ara-C or [5-$^3$H]NHAC (25 μCi per sample) for different time periods at 37°C (5% carbon dioxide). After washing twice with cold PBS, DNA was extracted as described by Spriggs *et al.* (1987). For the quantification of incorporated drug, the DNA was collected by filtration through Whatman GF/C filters (Whatman, Maidstone, UK). The filters were extensively washed with cold ethanol and the amount of incorporated drug quantified by scintillation counting (Momparler *et al.*, 1990).

### Results

*Deamination*

Figure 1 shows the kinetics of deamination of liposomal NHAC and ara-C in human plasma (Figure 1a) and mouse liver microsomes (Figure 1b). NHAC was almost completely resistant to deamination in plasma, resulting in a 42-fold reduction in ara-U formation after 4 h incubation. Expressed as a percentage, 84% of ara-C was deaminated to ara-U, whereas only 2% of NHAC was deaminated to ara-U. In the concentration-dependent study using freshly prepared mouse liver microsomes, NHAC was deaminated to ara-U at a slower rate and at concentrations 5–10 times lower than ara-C. These results demonstrate that NHAC is highly resistant to deamination.

**Figure 1** Deamination kinetics of ara-C and NHAC in human plasma (**a**) and mouse liver microsomes (**b**). (**a**) Fresh human plasma was exposed for various times to 2 μM ara-C (■) or NHAC (□). (**b**) Various concentrations of ara-C and NHAC were incubated with freshly prepared mouse liver microsomes. Symbols = mean from two separate experiments; bars = s.d. Where no error bars are seen, they are smaller than the size of the symbols.

*Cytotoxicity in HL-60 cells*

The cytotoxicity of NHAC and ara-C in HL-60 cells in the MTT dye reduction assay after a continuous 24 h incubation is shown in Figure 2a. As a negative control, the cytotoxicity of empty liposomes without NHAC was determined. While empty liposomes were not toxic to HL-60 cells at a lipid concentration up to 0.4 mg $ml^{-1}$ SPC, corresponding to a drug concentration in the drug-containing liposomes of 100 μM NHAC, further increase in the lipid concentration to 0.8 mg $ml^{-1}$ SPC (corresponding to liposomes with 200 μM NHAC) led to a weak toxicity of the empty liposomes, as demonstrated by a decrease in cell viability to 93% compared with the untreated control cells. The cytotoxic effect of NHAC-liposomes resulted in an $IC_{50}$ value of 47.0 ± 6.2 μM and an $IC_{20}$ value of 13.2 ± 2.7 μM respectively. Ara-C was more toxic than NHAC at low drug concentrations up to 20 μM with an $IC_{20}$ of 1 μM, whereas the $IC_{50}$ value was not reached in the concentration range up to 200 μM during a 24 h drug exposure. Therefore for ara-C the $IC_{20}$ values are given instead of the $IC_{50}$ values.

Figure 2b–d shows the cytotoxicity assays of ara-C and NHAC in HL-60 cells in combination with dipyridamole (Figure 2b), dCyd (Figure 2c) and dipyridamole plus dCyd (Figure 2d). Dipyridamole is a well-characterised nucleoside transporter-blocking agent (King *et al.*, 1984). As shown in Figure 2b, the cytotoxicity of ara-C was strongly reduced by dipyridamole ($IC_{20}$ = 215 ± 13 μM and $IC_{50}$>400 μM), but not with NHAC ($IC_{20}$ = 9.3 ± 1.3 μM and $IC_{50}$ = 48.9 ± 3.1 μM), indicating a nucleoside transporter-independent uptake mechanism for NHAC (see also Figure 4). dCyd is the physiological substrate of dCyd-kinase and a competitive inhibitor of ara-C phosphorylation (Coleman *et al.*, 1975). As shown in Figure 2c, the cytotoxicity of ara-C was significantly decreased by the addition of 20 μM dCyd 5 min before drug exposure. Thus, the $IC_{20}$ value for ara-C increased from 0.9 ± 0.1 μM to 186.3 ± 4.0 μM when the cells were pretreated with dCyd. In contrast, the cytotoxic effect of NHAC was not affected by dCyd, indicating that the mode of action of NHAC is independent to a great extent of the phosphorylation pathway. The combination of dipyridamole plus dCyd led to a further decrease in the cytotoxic effect of ara-C. As shown in Figure 2d, an $IC_{20}$ value for ara-C was therefore not reached at drug concentrations below 200 μM. In contrast, the cytotoxicity of NHAC was not influenced by pretreating the HL-60 cells with dipyridamole plus dCyd, resulting in an $IC_{50}$ value of 46.9 ± 3.4 μM, which is identical to the $IC_{50}$ value of the NHAC treatment without additives (Figure 2a) or the treatment with dipyridamole or dCyd alone (Figure 2b and c).

*Cytotoxicity in ara-C-resistant HL-60 cells (HL-60/ara-C)*

Figure 3 shows the concentration-dependent cytotoxic effects of ara-C and liposomal NHAC on the HL-60/ara-C-resistant cells. However, it should be noted that direct comparison of the HL-60 and HL-60/ara-C cell lines is difficult because of their different growth characteristics. The doubling time was found to be 30 h for the HL-60 cells and 22 h for the HL-60/ara-C cells. NHAC was more cytotoxic than ara-C to HL-60/ara-C cells in the concentration range up to 200 μM. However, compared with the ara-C-sensitive HL-60 cells the cytotoxic effect of NHAC was slightly reduced, requiring 77.0 ± 2.9 μM to reach the $IC_{50}$ as compared with 47.0 ± 6.2 μM for the sensitive HL-60 cells and 15.4 ± 2.3 μM to reach the $IC_{20}$ as compared with 13.2 ± 2.7 μM. Ara-C, on the other hand, was, as expected, significantly less cytotoxic to resistant HL-60/ara-C cells, not reaching an $IC_{50}$ value below 200 μM and resulting in a $IC_{20}$ of 52.2 ± 4.7 μM compared with 0.9 ± 0.1 μM for the sensitive HL-60 cells (Figure 2a).

*Cellular drug uptake*

Figure 4 summarises the cellular uptake of ara-C and liposomal NHAC in HL-60 cells in the presence and absence of the nucleoside transporter-blocking agent dipyridamole. The uptake of ara-C was slightly higher than that of NHAC at low drug concentrations up to 10 μM, however it was highly sensitive to dipyridamole. Thus, by adding dipyridamole 5 min before drug exposure, the uptake of ara-C decreased by a factor of 2.5–28 depending on the drug concentration. The uptake of NHAC, in contrast, was not blocked by dipyridamole, resulting in a 4- to 15-fold higher drug accumulation over the whole concentration range of 1–200 μM, and in a 1.5- to 6-fold higher uptake compared with ara-C alone in the range 20–200 μM. In contrast to NHAC, the uptake of ara-C revealed typical Michaelis–Menten kinetics, reaching saturation at 40–60 μM. These results provide evidence that the uptake of lipophilic NHAC is not dependent on the nucleoside transport mechanism and the number of transport sites per cell.

*Cellular pharmacokinetics*

Table I summarises the parameters of the intracellular pharmacokinetics of ara-C, NHAC and ara-CTP determined by incubating the cells for various periods in drug-free medium after 2 h drug exposure. All three compounds followed first-order kinetics during incubation periods of up to 3.5 h. The intracellular half-life for ara-C was 1.55 h, whereas the half-life for NHAC was 4.8 times longer, indicating a depot effect of the drug in the cellular membranes. The half-life for ara-CTP formed from NHAC was 1.8 times longer than that from ara-C. The longer half-life of ara-CTP originating from NHAC may be caused by the slow dealkylation of NHAC to ara-C and its subsequent phosphorylation to ara-CTP. The

**Figure 2** Cytotoxicity assays by MTT dye reduction in HL-60 cells. Cells were incubated with ara-C (■), NHAC-liposomes (□) or empty liposomes (*) for 24 h at 37°C (5% carbon dioxide) at a concentration range from 1 to 200 µM (**a**). (**b**) The cells were additionally incubated with 20 µM dipyridamole for 5 min before drug exposure. (**c**) dCyd 20 µM was added and in **d** a combination of 20 µM dipyridamole and 20 µM dCyd was used 5 min before drug exposure. Symbols = mean from four separate experiments; bars = s.d.

**Figure 3** Cytotoxicity assays by MTT dye reduction in the ara-C-resistant HL-60/ara-C cells. Cells were incubated with ara-C (■) or NHAC-liposomes (□) for 24 h at 37°C (5% carbon dioxide) at a concentration range from 1 to 200 µM. Symbols = mean from four separate experiments; bars = s.d.

**Figure 4** Uptake of ara-C and NHAC in HL-60 cells. Cells were exposed to various concentrations of ara-C or NHAC for 3 h at 37°C (5% carbon dioxide) in the presence or absence of the nucleoside transporter-blocking agent dipyridamole (20 µM). Dipyridamole was added 5 min before drug exposure. Ara-C (■), ara-C plus dipyridamole (●), NHAC (□), NHAC plus dipyridamole (○); Symbols = mean from three separate experiments; bars = s.d. Where no error bars are seen, they are smaller than the size of the symbols.

AUC of NHAC was 5.9 times that of ara-C, whereas the AUC of ara-CTP formed from ara-C was 3.6 times greater than formed from NHAC. Phosphorylated metabolites of NHAC (e.g. NHAC-triphosphate) were not detected by the method used for the measurement of ara-CTP.

*Ara-CTP formation*

The concentration-dependent formation of ara-CTP from ara-C and liposomal NHAC is shown in Figure 5. Significantly higher amounts of ara-CTP were formed from ara-C, reaching levels 5–90-times higher than those formed from NHAC. In contrast to NHAC, ara-C again revealed Michaelis–Menten kinetics, reaching saturation at 40 µM, whereas ara-CTP formation from NHAC increased with linear kinetics, not reaching saturation below 200 µM. The molar ratios of ara-CTP formed by ara-C and NHAC were for 1 µM and 20 µM drug concentration 90 and 39 respectively. In the concentration range of 1–60 µM, which is cytotoxic to HL-60 cells (Figure 2a), the amounts of ara-CTP formed from NHAC were 90 to 16 times lower. These low ara-CTP concentrations are not likely to be sufficient to

**Table I** Parameters of intracellular pharmacokinetics of ara-C, NHAC and ara-CTP in HL-60 cells

| *Determination* | *Drug*[a] | *Total drug* | *Ara-CTP* |
|---|---|---|---|
| Peak drug concentration[b] | Ara-C | 4.0±0.2[c] | 3.3±0.1[c] |
| (pmol $10^{-6}$ cells) | NHAC | 14.0±1.0 | 1.9±0.1 |
| Intracellular half-life[d] | Ara-C | 1.55±0.11[c] | 1.51±0.21[c] |
| (h) | NHAC | 7.42±0.58 | 2.69±0.25 |
| AUC[e] | Ara-C | 7.58 | 5.14 |
| (pmol $10^{-6}$ cells h) | NHAC | 44.66 | 1.41 |

[a]Ara-C was given in PBS solution and NHAC as a liposomal preparation. [b]Peak drug concentrations were determined after 2 h incubation with 2 μM ara-C or NHAC in HL-60 cells. [c]Mean±s.d. from three determinations. [d]Intracellular half-lives of ara-C, NHAC and ara-CTP in HL-60 cells after incubation with 2 μM ara-C or NHAC for 2 h. After incubation, the cells were washed to remove unbound drug and the incubation continued for various times up to 3.5 h. [e]Area under the curve (AUC) as calculated from three determinations fitted by first-order kinetics. Cells were incubated for 2 h with 2 μM ara-C or NHAC, washed to remove unbound drug and incubated in drug-free medium up to 3.5 h.

**Table II** DNA incorporation after incubation with ara-C or NHAC[a]

| | *DNA-incorporated drug (pmol $10^{-6}$ cells)* | | | |
|---|---|---|---|---|
| *Drug* | *1 h* | *3 h* | *6 h* | *24 h* |
| Ara-C[b] | 1.73 | 2.91 | 3.44 | 0.56 |
| NHAC[c] | 0.07 | 0.11 | 0.16 | 0.16 |

[a]DNA incorporation of metabolites formed after incubation of HL-60 cells with 2 μM ara-C or NHAC for various times. [b]Ara-C in PBS solution. [c]NHAC in liposomes.

contribute significantly to the cytotoxic activity of NHAC, as shown in the MTT dye reduction assays (Figure 2a and c).

### *Incorporation into DNA*

As shown in Table II, incorporation of metabolites into DNA after incubation with ara-C for up to 6 h was independent of the incubation time and on average 25 times higher than after NHAC incubation. After 24 h however, the incorporation into DNA of metabolites originating from ara-C was reduced, which can be explained by the higher cytotoxic effect on the HL-60 cells of ara-C at the concentration of 2 μM (Figure 2a).

### Discussion

In the present study, the cellular pharmacology of a liposomal preparation of NHAC, a new lipophilic alkyl derivative of ara-C, was investigated. Since the $N^4$-acyl-ara-C derivatives, including $N^4$-behenoyl-ara-C, have been considered as prodrugs which are activated through metabolisation to ara-C followed by phosphorylation to ara-CTP (Ueda *et al.*, 1983), we first studied whether the mechanism of cytotoxicity of the $N^4$-alkyl-ara-C derivative NHAC is the same as that of other cytidine analogues. The effects of ara-C on DNA synthesis and thus its cytotoxic activity are generally explained by two mechanisms. In the first, the cytotoxic effect is produced by inhibition of DNA polymerase by ara-CTP through enzyme competition with endogenous deoxy-CTP (Furth and Cohen, 1968; Momparler *et al.*, 1990). The incorporation of ara-CTP into DNA as a strand break-inducing nucleotide is the second mechanism and does not exclude the inhibition of DNA polymerase. Kufe and collaborators (Major *et al.*, 1981; Kufe *et al.*, 1984) have demonstrated in their studies that the extent of ara-C incorporation into DNA is the most powerful predictor of cytotoxicity.

Our results with NHAC indicate, however, that although small amounts of ara-C and ara-CTP are derived from NHAC (Figure 5), these low concentrations of ara-CTP and DNA-incorporated metabolites (Tables I and II) are unlikely

**Figure 5** Ara-CTP formation in HL-60 cells. Cells were exposed to various concentrations of ara-C (■) or NHAC (□) for 2 h at 37°C (5% carbon dioxide). Ara-CTP concentration was determined by HPLC analysis of cell lysate supernatants. Symbols = mean from three separate experiments; bars = s.d.

to be exclusively responsible for the cytotoxic effects of NHAC in HL-60 cells. The fact that NHAC was still cytotoxic in the ara-C-resistant HL-60/ara-C cells, requiring only a 2-fold higher drug concentration to reach the $IC_{50}$ value, provides further evidence that other mechanisms of action are responsible for the cytotoxic activity of NHAC. The maintenance of the cytotoxic activity in the ara-C-resistant cells indicates that NHAC might still be active in ara-C-resistant leukaemias in which high activity of cytidine deaminase, decreased numbers of nucleoside transport sites, low deoxycytidine kinase activity and high deoxycytidine triphosphate pools are causing ara-C resistance (Steuart and Burke 1971; Tattersall *et al.*, 1974).

The cellular uptake of NHAC could not be saturated at drug concentrations up to 200 μM and was again, in contrast to ara-C, independent of the number of nucleoside transport sites, suggesting that drug uptake is achieved by a passive diffusion process mainly resulting from the lipophilic properties of NHAC rather than by a transporter-mediated, active mechanism. Thus, the uptake of NHAC in leukaemia cells might be explained by fusion of liposomes with the cellular membranes, by direct transfer of NHAC from the liposomes to the cell membranes or by liposome phagocytosis.

Probably the most significant advantage of NHAC over ara-C consists in its greatly improved stability against deamination, which might be responsible for the difference in cellular pharmacological properties such as the longer intracellular half-life (Table I). These properties allow NHAC to be administered at lower doses than ara-C to obtain better anti-tumour effects *in vivo* (Schwendener and Schott, 1992). The possibility of producing large batches of sterile and stable drug-containing liposomes (Schwendener, 1992) provides the opportunity to enter clinical studies with NHAC. Owing to the high lipophilicity of the NHAC molecule, it is essential to incorporate NHAC into liposomes in order to obtain a physiological drug formulation. The liposomes themselves and their size have no significant effect on the uptake of NHAC *in vitro* (data not shown). The release of NHAC from the liposomes and its interactions with blood components have been studied in detail and the results will be reported separately (Horber *et al.*, 1995).

In conclusion, our study strongly suggests that NHAC is able to overcome ara-C resistance by circumvention of the major reasons for treatment failure among leukaemia patients. Thus, NHAC is cytotoxic independently of the nucleoside transport mechanism and the dCyd kinase activity in HL-60 cells and it still retains its activity in ara-C-resistant HL-60/ara-C cells. Furthermore, the cellular uptake of liposomal NHAC is higher than that of ara-C and independent of the nucleoside transport mechanism. Long-chain alkylation of the amino group of ara-C provides excellent protection against deamination and inactivation to ara-U. Although the exact mechanism of action has not yet been elucidated and further pharmacological studies are required, nevertheless it seems to be obvious that the mechanism of cytotoxicity of NHAC is very different from that of ara-C or the $N^4$-acyl-ara-C derivatives.

### Acknowledgments

The authors wish to thank K Rentsch for the protein determinations. This work was supported by the Sassella Foundation, Stiftung für Krebsbekämpfung, the Stiftung für angewandte Krebsforschung and the Swiss National Science Foundation (Grant No. 32-29979.90).

### References

AOSHIMA M, TSUKAGOSHI S, SAKURAI Y, OH-ISHI J AND ISHIDA T. (1976). Antitumour activities of newly synthesized $N^4$-acyl-1-β-D-arabinofuranosylcytosine. *Cancer Res.*, **36**, 2726–2732.

BHALLA K, NAYAK R AND GRANT S. (1984). Isolation and characterization of a deoxycytidine kinase-deficient human promyelocytic leukemic cell line highly resistant to 1-β-D-arabinofuranosylcytosine. *Cancer Res.*, **44**, 5029–5037.

BRADFORD MM. (1976). A rapid and sensitive method for the quantification of microgram quantities of protein utilizing the principle of protein–dye binding. *Anal. Biochem.*, **72**, 248–254.

CHU MY AND FISHER GA. (1965). Comparative studies of leukemic cells sensitive and resistant to cytosine arabinoside. *Biochem. Pharmacol.*, **14**, 333–341.

COLEMAN CN, STOLLER RG, DRAKE JC AND CHABNER BA. (1975). Deoxycytidine kinase: properties of the enzyme from human leukemic granulocytes. *Blood*, **46**, 791–803.

DRISCOLL JS, MARQUEZ VE, PLOWMAN J, LIU PS, KELLEY JA AND BARCHI JJ. (1991). Antitumour properties of 2(1H)-pyrimidinone riboside (zebularine) and its fluorinated analogues. *J. Med. Chem.*, **34**, 3280–3284.

FREI EI, BICKERS JN, HEWLETT JS, LANE M, LEARY WV AND TALLEY RW. (1969). Dose schedule and antitumour studies of arabinosyl cytosine. *Cancer Res.*, **29**, 1325–1332.

FURTH JJ AND COHEN SS. (1968). Inhibition of mammalian DNA polymerase by 5′-triphosphate of 1-β-D-arabinofuranosylcytosine and the triphosphate of 1-β-D-arabinofuranosylcytosine. *Cancer Res.*, **28**, 2061–2067.

HO DH AND FREI EI. (1971). Clinical pharmacology of 1-β-D-arabinofuranosylcytosine. *Clin. Pharmacol. Ther.*, **12**, 944–954.

HOPE MJ, BALLY MB, WEBB G AND CULLIS PR. (1985). Production of large unilamellar vesicles by a rapid procedure, characterization of size distribution, trapped volume and ability to maintain a membrane potential. *Biochem. Biophys. Acta*, **812**, 55–65.

HORBER DH, OTTIGER C, SCHOTT H AND SCHWENDENER RA. (1995). Pharmakokinetic properties and interactions with blood components of $N^4$-hexadecyl-1-β-D-arabinofuranosylcytosine (NHAC) incorporated into liposomes. *J. Pharm. Pharmacol.* (in press).

HOULIHAN WJ, LOHMEYER M, WORKMAN P AND CHEON SH. (1995). Phospholipid antitumour agents. *Med. Res. Reviews.* (in press).

KANAI T AND ICHINO M. (1974). Pyrimidine nucleosides. 6. Syntheses and anticancer activities of $N^4$-substituted 2,2′-anhydronucleosides. *J. Med. Chem.*, **17**, 1076–1078.

KATAOKA T AND SAKURAI Y. (1980). Effect and mode of action of $N^4$-behenoyl-β-D-arabinofuranosylcytosine. *Recent Results Cancer Res.*, **70**, 147–151.

KEATING MJ, McCREDIE KB, BODEY GP, SMITH TL, GEHAN E AND FREIREICH EJ. (1982). Improved prospects for long term survival in adults with acute myelogenous leukemia. *J. Am. Med. Assoc.*, **248**, 2481–2486.

KING ME, NAPORN A, YOUNG B AND HOWELL SB. (1984). Modulation of cytarabine uptake and toxicity by dipyridamole. *Cancer Treat. Reports*, **68**, 361–366.

KOLLA SS AND STUDZINSKI GP. (1994). Constitutive DNA binding of the low mobility forms of the AP-1 and SP-1 transcription factors in HL-60 cells resistant to 1-β-D-arabinofuranosylcytosine. *Cancer Res.*, **54**, 1418–1421.

KREIS W, BUDMAN DR, CHAN K, ALLEN SL, SCHULMAN P, LICHTMAN S, WEISELBERG L, SCHUSTER M, FREEMAN J, AKERMAN S, ATTAS L AND VINCIGUERRA V. (1991). Therapy of refractory/relapsed acute leukemia with cytosine arabinoside plus tetrahydrouridine (an inhibitor of cytidine deaminase) – a pilot study. *Leukemia*, **5**, 991–998.

KUFE D, SPRIGGS D, EGAN EM AND MUNROE D. (1984). Relationships among Ara-CTP pools, formation of (Ara-C)DNA, and cytotoxicity of human leukemic cells. *Blood*, **64**, 54–58.

MAJOR PP, EGAN EM, BEARDSLEY GP, MINDEN MD AND KUFE DW. (1981). Lethality of human myeloblasts correlates with the incorporation of arabinofuranosylcytosine into DNA. *Proc. Natl Acad. Sci. USA*, **78**, 3235–3239.

MOMPARLER RL. (1974). A model for the chemotherapy of acute leukemia with 1-β-D-arabinofuranosylcytosine. *Cancer Res.*, **34**, 1775–1787.

MOMPARLER RL, ONETTO PN, BOUFFARD DY AND MOMPARLER LF. (1990). Cellular pharmacology of 1-β-D-arabinofuranosylcytosine in human myeloid, B-lymphoid and T-lymphoid leukemic cells. *Cancer Chemother. Pharmacol.*, **27**, 141–146.

MOSMANN T. (1983). Rapid colorimetric assay for cellular growth and survival: application to proliferation and cytotoxicity assays. *J. Immunol. Methods*, **65**, 55–63.

PLAGEMANN PG, MARZ R AND WOHLHUETER RM. (1978). Transport and metabolism of deoxycytidine and 1-β-D-arabinofuranosylcytosine into cultured Novikoff rat hepatoma cells, relationship to phosphorylation and regulation of triphosphate synthesis. *Cancer Res.*, **38**, 978–989.

PLUNKETT W AND GANDHI V. (1993). Cellular pharmacodynamics of anticancer drugs. *Semin. Oncol.*, **20**, 50–63.

ROSOWSKY A, KIM SH, ROSS J AND WICK MM. (1982). Lipophilic 5′-(alkyl phosphate) esters of 1-β-D-arabinofuranosylcytosine and its $N^4$-acyl and 2,2′-anhydro-3′-O-acyl derivatives as potential prodrugs. *J. Med. Chem.*, **25**, 171–178.

RUBAS W, SUPERSAXO A, WEDER HG, HARTMANN HR, HENGARTNER H, SCHOTT H AND SCHWENDENER R. (1986). Treatment of murine L1210 lymphoid leukemia and melanoma B16 with lipophilic cytosine arabinoside prodrugs incorporated into unilamellar liposomes. *Int. J. Cancer*, **37**, 149–154.

SCHWENDENER RA. (1992). The preparation of large volumes of sterile liposomes for clinical applications. In: *Liposome Technology*, 2nd edn, Gregoriadis G (ed.) pp. 487–500. CRC Press: Boca Raton FL.

SCHWENDENER RA AND SCHOTT H. (1992). Treatment of L1210 murine leukemia with liposome-incorporated $N^4$-hexadecyl-1-β-D-arabinofuranosylcytosine. *Int. J. Cancer*, **51**, 466–469.

SCHWENDENER RA, PESTALOZZI B, BERGER S, SCHOTT H, HENGARTNER H AND SAUTER C. (1989). Treatment of acute myelogenous leukemia with liposomes containing $N^4$-oleylcytosine arabinoside. In: *Liposomes in the Therapy of Infectious Diseases and Cancer*, Lopez-Berenstein G and Fidler IJ (eds.) pp. 95–103. Alan R Liss, Inc.: New York, NY.

SPRIGGS D, ROBBINS G, OHNO Y AND KUFE D. (1987). Detection of 1-β-D-arabinofuranosylcytosine incorporation into DNA *in vivo*. *Cancer Res.*, **47**, 6532–6536.

STEUART CD AND BURKE PJ. (1971). Cytidine deaminase and the development of resistance to arabinosylcytosine. *Nature New Biol.*, **233**, 109–110.

TATTERSALL MH, GANESHAGURU K AND HOFFBRAND AV. (1974). Mechanisms of resistance of human acute leukaemia cells to cytosine arabinoside. *Br. J. Haematol*, **27**, 39–46.

TSURUO T, TSUKAGOSHI S AND SAKURAI Y. (1980). $N^4$-palmitoyl- and $N^4$-stearoyl-1-β-D-arabinofuranosylcytosine as new antitumour agents. In *Current Chemotherapy and Infectious Disease*, Grassi JD. (ed.) pp. 1591–1593. American Society of Microbiology: Washington DC.

UEDA T, NAKAMURA T, KAGAWA D, YAMAMOTO K, UCHIDA A, SASADA M AND UCHINO H. (1983). Intracellular distribution of $N^4$-behenoyl-1-β-D-arabinofuranosylcytosine in blood cells. *Gann*, **74**, 445–451.

WEMPEN I, MILLER N, FALCO EA AND FOX JJ. (1968). Nucleosides. XLVII. Synthesis of some $N^4$-substituted derivatives of 1-β-D-arabinofuranosylcytosine and 5-fluorocytosine. *J. Med. Chem.*, **11**, 144–148.

WILEY JS, JONES SP, SAWYER WH AND PATERSON AR. (1982). Cytosine arabinoside influx and nucleoside transport sites in acute leukemia. *J. Clin. Invest.*, **69**, 479–489.


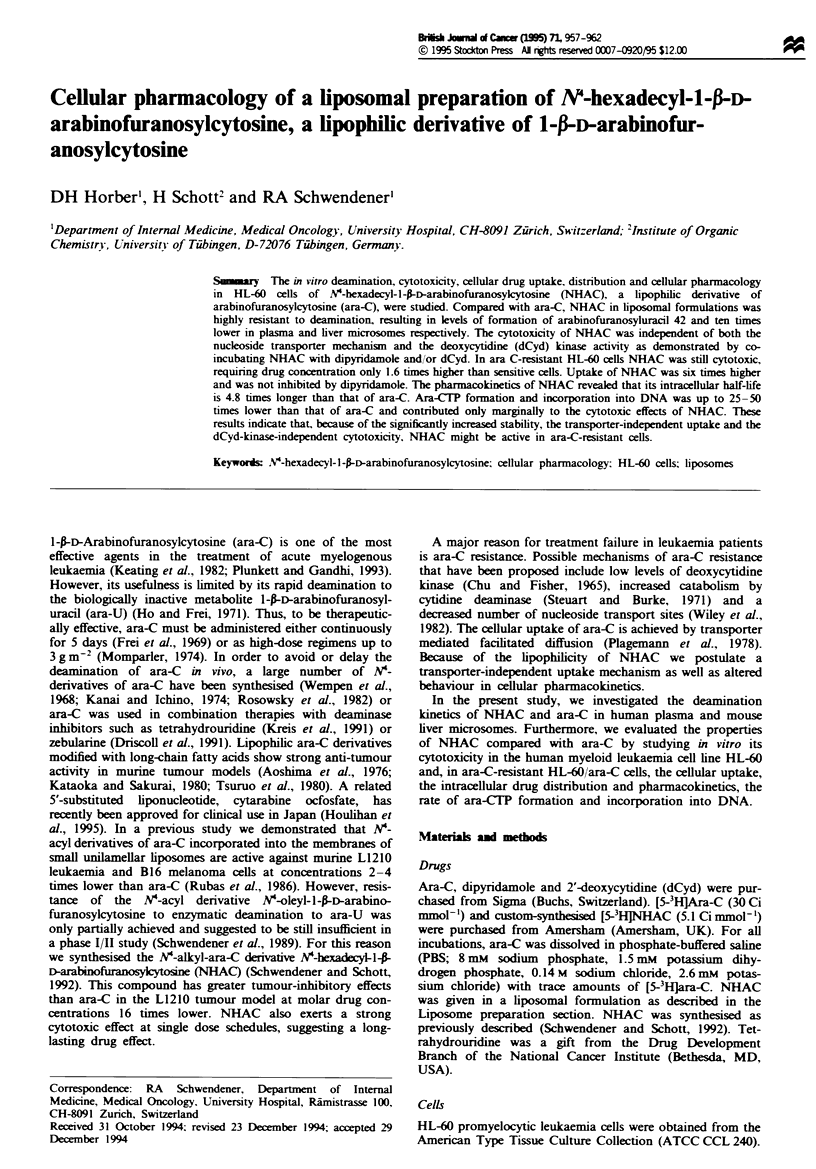

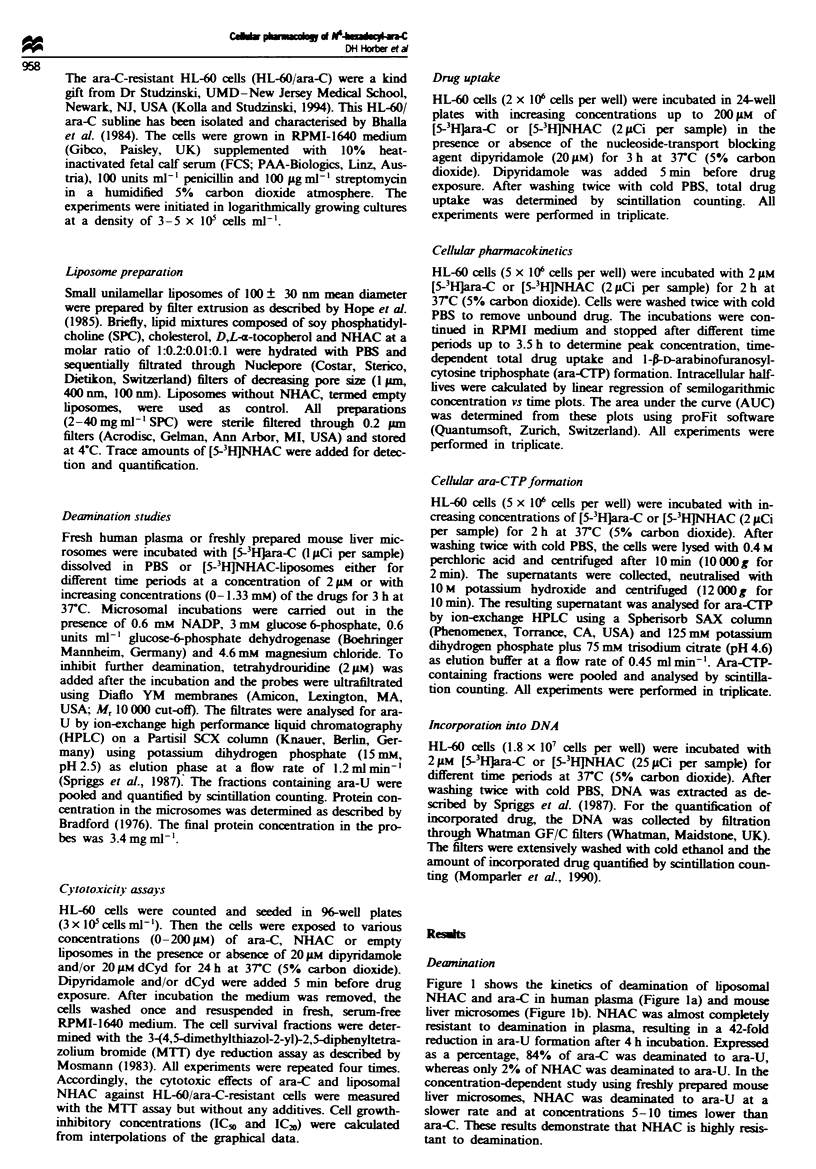

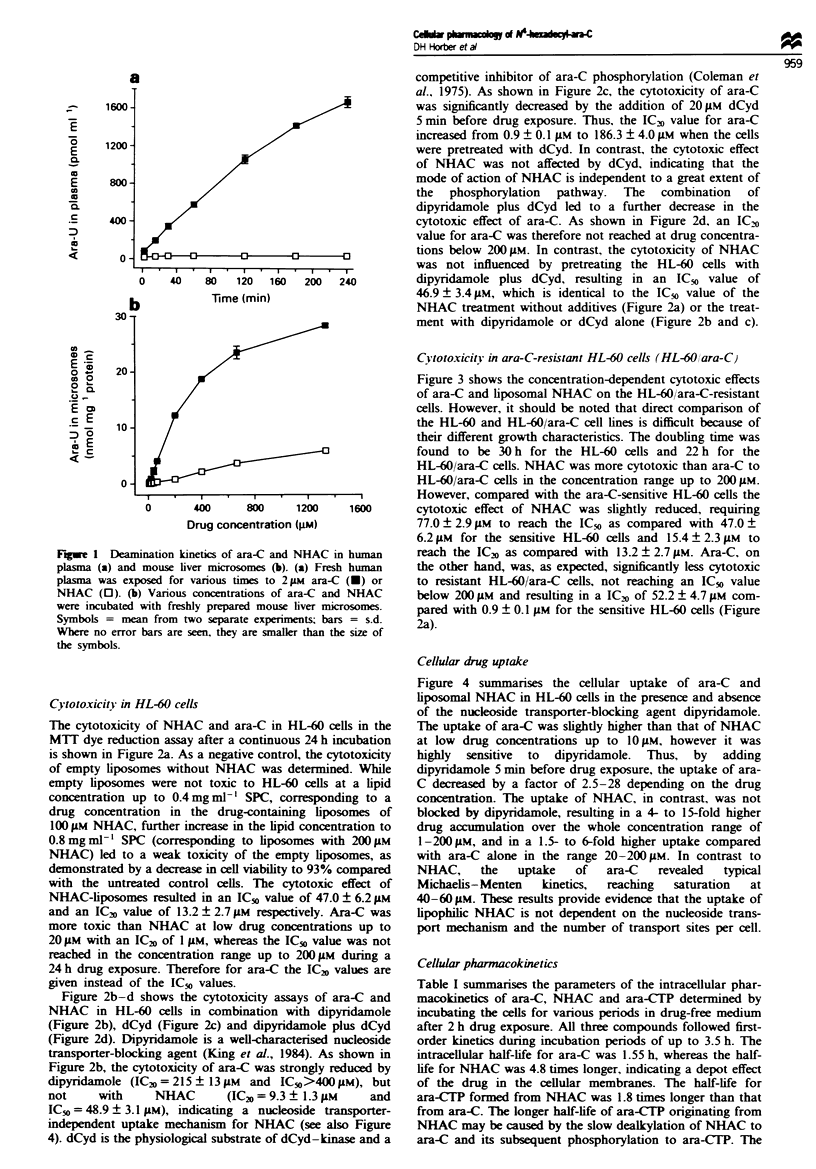

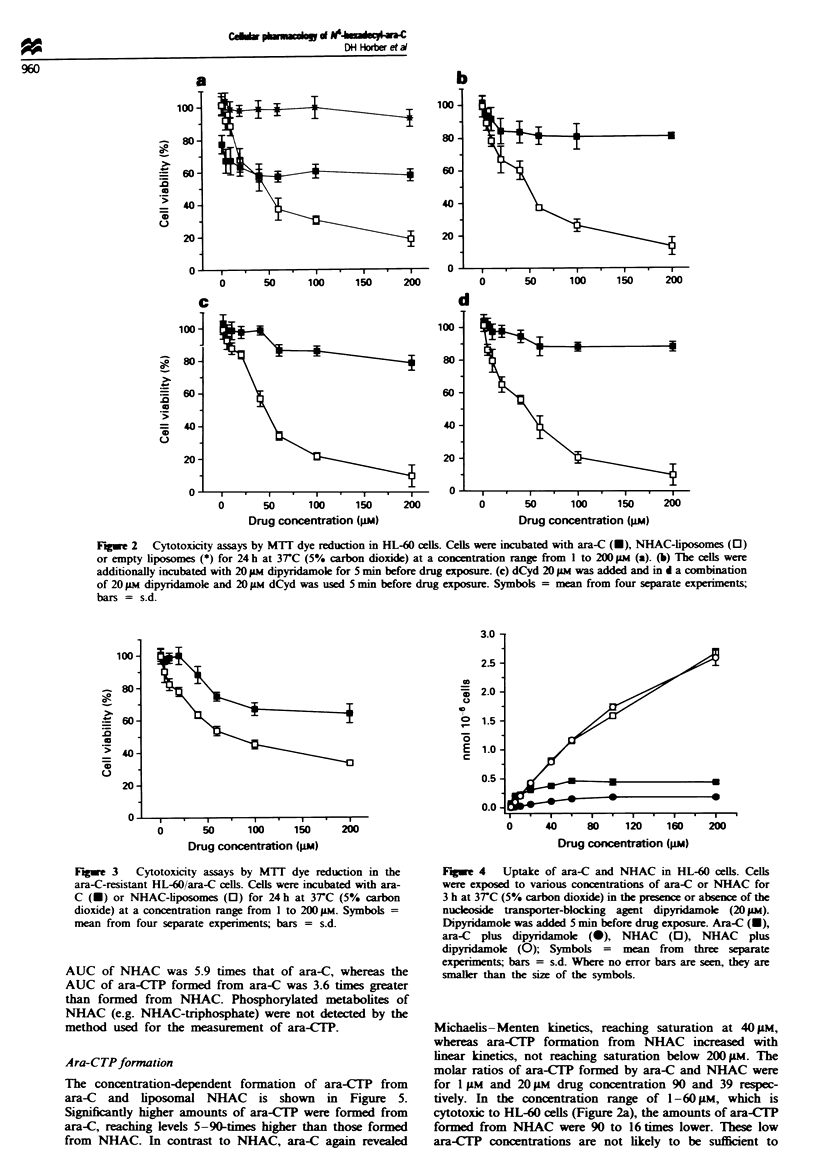

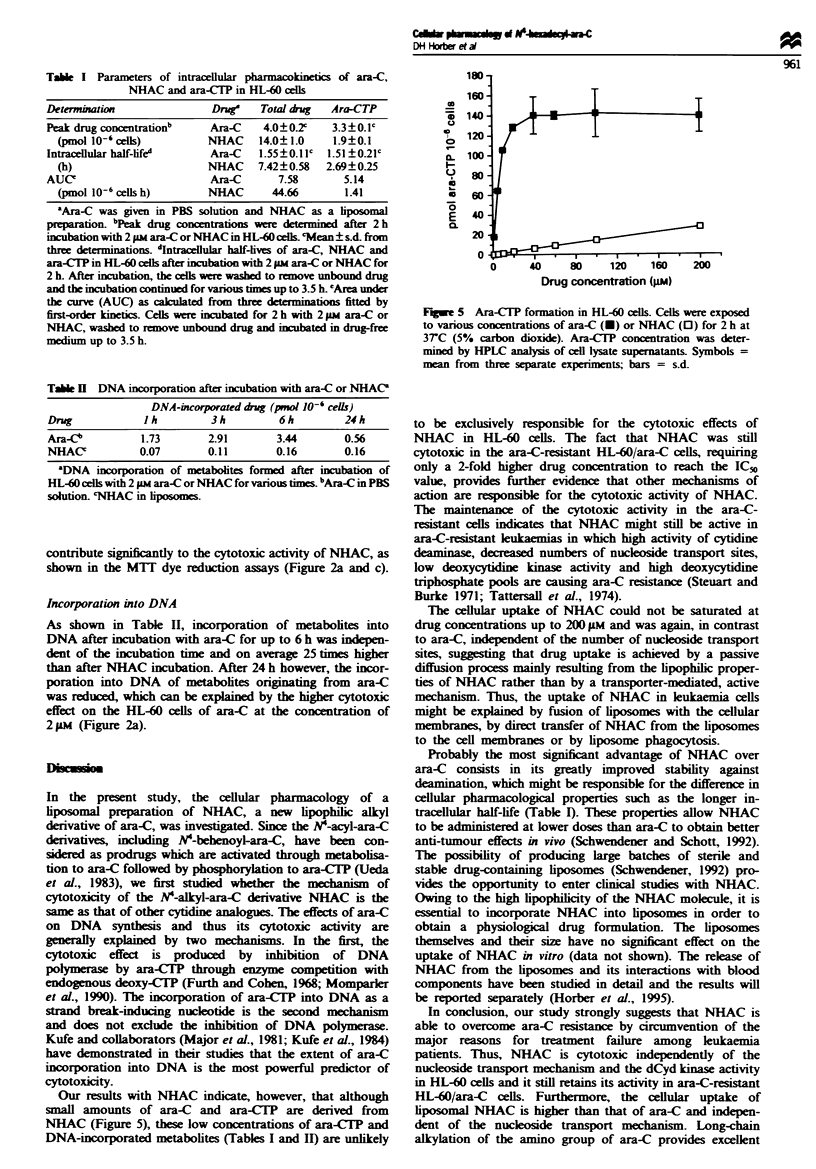

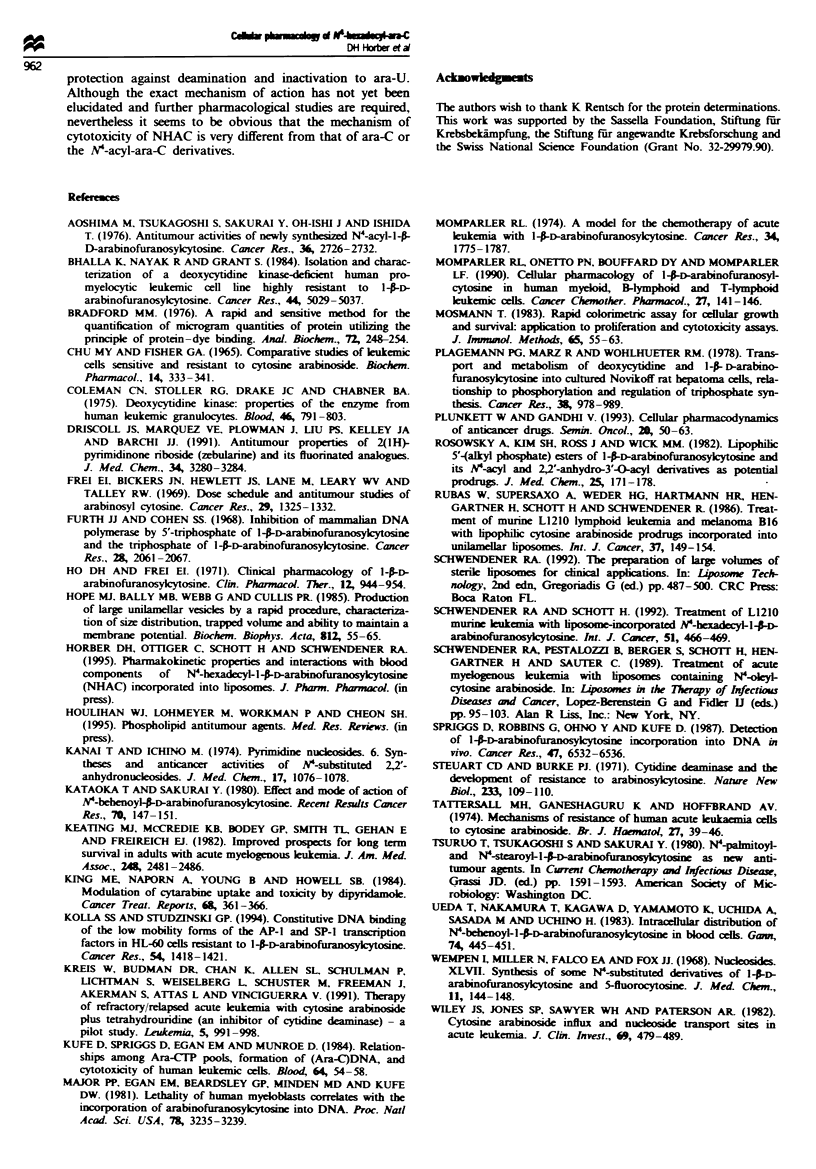


## References

[OCR_00848] Aoshima M., Tsukagoshi S., Sakurai Y., Oh-ishi J., Ishida T. (1976). Antitumor activities of newly synthesized N4-acyl-1-beta-D-arabinofuranosylcytosine.. Cancer Res.

[OCR_00853] Bhalla K., Nayak R., Grant S. (1984). Isolation and characterization of a deoxycytidine kinase-deficient human promyelocytic leukemic cell line highly resistant to 1-beta-D- arabinofuranosylcytosine.. Cancer Res.

[OCR_00857] Bradford M. M. (1976). A rapid and sensitive method for the quantitation of microgram quantities of protein utilizing the principle of protein-dye binding.. Anal Biochem.

[OCR_00861] CHU M. Y., FISCHER G. A. (1965). COMPARATIVE STUDIES OF LEUKEMIC CELLS SENSITIVE AND RESISTANT TO CYTOSINE ARABINOSIDE.. Biochem Pharmacol.

[OCR_00866] Coleman C. N., Stoller R. G., Drake J. C., Chabner B. A. (1975). Deoxycytidine kinase: properties of the enzyme from human leukemic granulocytes.. Blood.

[OCR_00874] Driscoll J. S., Marquez V. E., Plowman J., Liu P. S., Kelley J. A., Barchi J. J. (1991). Antitumor properties of 2(1H)-pyrimidinone riboside (zebularine) and its fluorinated analogues.. J Med Chem.

[OCR_00877] Frei E., Bickers J. N., Hewlett J. S., Lane M., Leary W. V., Talley R. W. (1969). Dose schedule and antitumor studies of arabinosyl cytosine (NSC 63878).. Cancer Res.

[OCR_00884] Furth J. J., Cohen S. S. (1968). Inhibition of mammalian DNA polymerase by the 5'-triphosphate of 1-beta-d-arabinofuranosylcytosine and the 5'-triphosphate of 9-beta-d-arabinofuranoxyladenine.. Cancer Res.

[OCR_00888] Ho D. H., Frei E. (1971). Clinical pharmacology of 1-beta-d-arabinofuranosyl cytosine.. Clin Pharmacol Ther.

[OCR_00909] Kanai T., Ichino M. (1974). Pyrimidine nucleosides. 6. Syntheses and anticancer activities of N4-substituted 2,2'-anhydronucleosides.. J Med Chem.

[OCR_00914] Kataoka T., Sakurai Y. (1980). Effect and mode of action of N4-behenoyl-beta-D-arabinofuranosylcytosine.. Recent Results Cancer Res.

[OCR_00922] Keating M. J., McCredie K. B., Bodey G. P., Smith T. L., Gehan E., Freireich E. J. (1982). Improved prospects for long-term survival in adults with acute myelogenous leukemia.. JAMA.

[OCR_00927] King M. E., Naporn A., Young B., Howell S. B. (1984). Modulation of cytarabine uptake and toxicity by dipyridamole.. Cancer Treat Rep.

[OCR_00930] Kolla S. S., Studzinski G. P. (1994). Constitutive DNA binding of the low mobility forms of the AP-1 and SP-1 transcription factors in HL60 cells resistant to 1-beta-D-arabinofuranosylcytosine.. Cancer Res.

[OCR_00939] Kreis W., Budman D. R., Chan K., Allen S. L., Schulman P., Lichtman S., Weiselberg L., Schuster M., Freeman J., Akerman S. (1991). Therapy of refractory/relapsed acute leukemia with cytosine arabinoside plus tetrahydrouridine (an inhibitor of cytidine deaminase)--a pilot study.. Leukemia.

[OCR_00944] Kufe D., Spriggs D., Egan E. M., Munroe D. (1984). Relationships among Ara-CTP pools, formation of (Ara-C)DNA, and cytotoxicity of human leukemic cells.. Blood.

[OCR_00951] Major P. P., Egan E. M., Beardsley G. P., Minden M. D., Kufe D. W. (1981). Lethality of human myeloblasts correlates with the incorporation of arabinofuranosylcytosine into DNA.. Proc Natl Acad Sci U S A.

[OCR_00955] Momparler R. L. (1974). A model for the chemotherapy of acute leukemia with 1-beta-D-arabinofuranosylcytosine.. Cancer Res.

[OCR_00960] Momparler R. L., Onetto-Pothier N., Bouffard D. Y., Momparler L. F. (1990). Cellular pharmacology of 1-beta-D-arabinofuranosylcytosine in human myeloid, B-lymphoid and T-lymphoid leukemic cells.. Cancer Chemother Pharmacol.

[OCR_00966] Mosmann T. (1983). Rapid colorimetric assay for cellular growth and survival: application to proliferation and cytotoxicity assays.. J Immunol Methods.

[OCR_00973] Plagemann P. G., Marz R., Wohlhueter R. M. (1978). Transport and metabolism of deoxycytidine and 1-beta-D-arabinofuranosylcytosine into cultured Novikoff rat hepatoma cells, relationship to phosphorylation, and regulation of triphosphate synthesis.. Cancer Res.

[OCR_00980] Plunkett W., Gandhi V. (1993). Cellular pharmacodynamics of anticancer drugs.. Semin Oncol.

[OCR_00984] Rosowsky A., Kim S. H., Ross J., Wick M. M. (1982). Lipophilic 5'-(alkyl phosphate) esters of 1-beta-D-arabinofuranosylcytosine and its N4-acyl and 2,2'-anhydro-3'-O-acyl derivatives as potential prodrugs.. J Med Chem.

[OCR_00990] Rubas W., Supersaxo A., Weder H. G., Hartmann H. R., Hengartner H., Schott H., Schwendener R. (1986). Treatment of murine L1210 lymphoid leukemia and melanoma B16 with lipophilic cytosine arabinoside prodrugs incorporated into unilamellar liposomes.. Int J Cancer.

[OCR_01003] Schwendener R. A., Schott H. (1992). Treatment of L1210 murine leukemia with liposome-incorporated N4-hexadecyl-1-beta-D-arabinofuranosyl cytosine.. Int J Cancer.

[OCR_01016] Spriggs D., Robbins G., Ohno Y., Kufe D. (1987). Detection of 1-beta-D-arabinofuranosylcytosine incorporation into DNA in vivo.. Cancer Res.

[OCR_01021] Steuart C. D., Burke P. J. (1971). Cytidine deaminase and the development of resistance to arabinosyl cytosine.. Nat New Biol.

[OCR_01026] Tattersall M. H., Ganeshaguru K., Hoffbrand A. V. (1974). Mechanisms of resistance of human acute leukaemia cells to cytosine arabinoside.. Br J Haematol.

[OCR_01038] Ueda T., Nakamura T., Kagawa D., Yamamoto K., Uchida M., Sasada M., Uchino H. (1983). Intracellular distribution of N4-behenoyl-1-beta-D-arabinofuranosylcytosine in blood cells.. Gan.

[OCR_01042] Wempen I., Miller N., Falco E. A., Fox J. J. (1968). Nucleosides. XLVII. Synthesis of some N4-substituted derivarives of 1-beta-D-arabinofuranosylcytosine and -5-fluorocytosine.. J Med Chem.

[OCR_01048] Wiley J. S., Jones S. P., Sawyer W. H., Paterson A. R. (1982). Cytosine arabinoside influx and nucleoside transport sites in acute leukemia.. J Clin Invest.

